# Chemical Screening Approaches Enabling Drug Discovery of Autophagy Modulators for Biomedical Applications in Human Diseases

**DOI:** 10.3389/fcell.2019.00038

**Published:** 2019-03-19

**Authors:** Prashanta Kumar Panda, Alexandra Fahrner, Somya Vats, Elena Seranova, Vartika Sharma, Miruna Chipara, Priyal Desai, Jorge Torresi, Tatiana Rosenstock, Dhiraj Kumar, Sovan Sarkar

**Affiliations:** ^1^Institute of Cancer and Genomic Sciences, College of Medical and Dental Sciences, University of Birmingham, Birmingham, United Kingdom; ^2^Molecular Biology and Genetics Unit, Jawaharlal Nehru Centre for Advanced Scientific Research, Bengaluru, India; ^3^Cellular Immunology Group, International Centre for Genetic Engineering and Biotechnology, New Delhi, India; ^4^Department of Physiological Science, Santa Casa de São Paulo School of Medical Sciences, São Paulo, Brazil

**Keywords:** autophagy, autophagy reporter, autophagy substrate, autophagy modulator, screening method, neurodegenerative diseases, cancer, lifespan extension

## Abstract

Autophagy is an intracellular degradation pathway for malfunctioning aggregation-prone proteins, damaged organelles, unwanted macromolecules and invading pathogens. This process is essential for maintaining cellular and tissue homeostasis that contribute to organismal survival. Autophagy dysfunction has been implicated in the pathogenesis of diverse human diseases, and therefore, therapeutic exploitation of autophagy is of potential biomedical relevance. A number of chemical screening approaches have been established for the drug discovery of autophagy modulators based on the perturbations of autophagy reporters or the clearance of autophagy substrates. These readouts can be detected by fluorescence and high-content microscopy, flow cytometry, microplate reader and immunoblotting, and the assays have evolved to enable high-throughput screening and measurement of autophagic flux. Several pharmacological modulators of autophagy have been identified that act either via the classical mechanistic target of rapamycin (mTOR) pathway or independently of mTOR. Many of these autophagy modulators have been demonstrated to exert beneficial effects in transgenic models of neurodegenerative disorders, cancer, infectious diseases, liver diseases, myopathies as well as in lifespan extension. This review describes the commonly used chemical screening approaches in mammalian cells and the key autophagy modulators identified through these methods, and highlights the therapeutic benefits of these compounds in specific disease contexts.

## Introduction

Macroautophagy, herein referred to as autophagy, is an intracellular degradation process essential for ensuring cellular homeostasis. This well-conserved catabolic process mediates the targeted degradation of unwanted or excess cytoplasmic materials, such as aggregation-prone proteins, pathogens and damaged organelles like mitochondria, amongst others (Ravikumar et al., 2010). This process is also involved in the bulk degradation of cytoplasmic macromolecules and recycling of the breakdown products especially during nutrient deprivation to provide energy homeostasis, thereby forming a crucial connection between anabolism and catabolism (Boya et al., 2013; Kaur and Debnath, 2015). Due to its vital function as a homeostatic regulator, impairment of the autophagy is implicated in several human pathologies including certain cancer, metabolic syndromes, infectious diseases, liver diseases, myopathies, aging and neurodegenerative disorders (Mizushima et al., 2008). Therefore, therapeutic modulation of autophagy holds great potential in the development of treatment strategies for these diseases (Rubinsztein et al., 2012).

**FIGURE 1:**
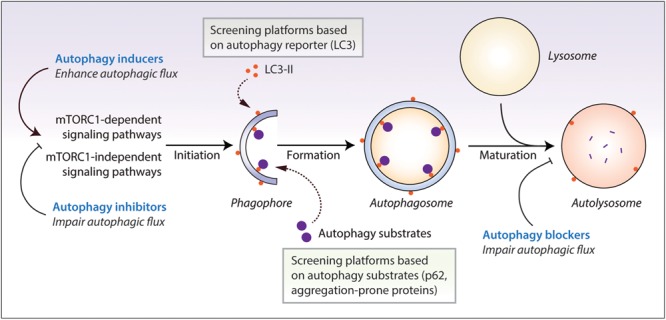
Autophagy reporter and substrate based screening strategies and the impact of autophagy modulators at different stages of the autophagy process. Autophagy is regulated by the mechanistic target of rapamycin complex 1 (mTORC1) or mTORC1-independent pathways. This process initiates by the formation of phagophores that expand and engulf autophagy substrates to form autophagosomes, which then fuse with the lysosomes to form autolysosomes where the autophagic cargo is degraded. Autophagy inducers and inhibitors increase or decrease autophagosome formation, respectively, at the early stages of autophagy, whereas autophagy blockers prevent lysosomal degradation and/or autophagosome maturation at late stages of autophagy. Autophagic flux is thus enhanced by autophagy inducers but is retarded by autophagy inhibitors and blockers. Chemical screening methods for identifying autophagy modulators are commonly based on the readouts of perturbations in autophagy reporters such as LC3-II, or autophagy substrate clearance such as aggregation-prone proteins or p62/SQSTM1.

Autophagy is evolutionarily-conserved from yeast to humans. The *de novo* formation of phagophores, the double-membrane structures that expand to form double-membrane vesicles called autophagosomes, require multiple autophagy-related (*Atg*) genes in the autophagic machinery, such as the Atg5-Atg12-Atg16 complex and the phosphatidylethanolamine-conjugated microtubule-associated protein 1 light chain 3 (LC3-II) (Kabeya et al., 2000; Mizushima et al., 2011; Ktistakis and Tooze, 2016). Maturation of autophagosomes into the degradative autolysosomes occurs either via the multi-step route involving the fusion of autophagosomes with late endosomes to form amphisomes which subsequently fuse with the lysosomes, or via the direct route involving the fusion between autophagosomes and the lysosomes (Nakamura and Yoshimori, 2017). The autophagic cargo engulfed by the autophagosomes are ultimately degraded in the acidic autolysosomes by the lysosomal hydrolases, which are only active at the low pH maintained by the vacuolar-type H^+^-ATPase (V-ATPase) on the lysosomal membrane (Saftig and Klumperman, 2009). Finally, the breakdown products are recycled and utilized as inputs to cellular metabolism for energy generation (Rabinowitz and White, 2010). The rate at which this dynamic turnover of cellular contents occurs through the process of autophagy is referred to as autophagic flux. Autophagic flux encompasses all stages of autophagy which includes autophagosome formation, fusion with the lysosomes and cargo degradation in the autolysosomes (Figure 1).

Key upstream modulators of autophagy include the mechanistic target of rapamycin complex 1 (mTORC1) pathway, which promotes cellular biosynthesis and inhibits autophagy (Saxton and Sabatini, 2017). Regulation of autophagosome formation by mTORC1 is mediated via the ULK1–Atg13–FIP200 complex; mTORC1 suppresses autophagy under nutrient-rich conditions by phosphorylation-dependent inactivation of ULK1 and Atg13 (Mizushima, 2010; Zachari and Ganley, 2017). Various signals such as growth factors and nutrients impinge on mTORC1 to negatively influence autophagy (Kim and Guan, 2015). Conversely, during nutrient starvation, autophagy is promoted by inhibition of the mTORC1 activity (Carroll et al., 2014; Russell et al., 2014). Furthermore, ULK1 can be directly phosphorylated and activated by the energy sensor AMPK to stimulate autophagy (Egan et al., 2011; Kim et al., 2011). In addition, several mTORC1-independent pathways have been described where autophagy is negatively regulated by the elevation in intracellular inositol, Ca^2+^ and nitric oxide levels, amongst others (Sarkar, 2013b). Molecular mediators of the late stage of autophagy involving autophagosome maturation include Rab7, SNAREs (N-ethylmaleimide-sensitive factor-attachment protein receptors), GABARAPs, BRUCE and Beclin1-interacting partners such as Atg14L, UVRAG and Ambra1 (He and Levine, 2010; Nguyen et al., 2016; Wang et al., 2016; Reggiori and Ungermann, 2017; Ebner et al., 2018). At a transcriptional level, autophagy is governed by the transcription factor EB (TFEB) (Settembre et al., 2011), which in itself is activated by lysosomal Ca^2+^ (Medina et al., 2015).

Chemical modulation of autophagy by targeting the mTOR-dependent and mTOR-independent pathways has proven to be of potential biomedical relevance due to therapeutic advantages, especially in neurodegenerative disorders as well as in diverse human pathological conditions such as in certain liver diseases, myopathies, infectious diseases, metabolic diseases, cancer and aging (Rubinsztein et al., 2012; Sarkar, 2013b; Levine et al., 2015). Hence, the discovery of potent small molecules regulating autophagy is of great interest. Here we review the chemical screening strategies for autophagy drug discovery, and highlight the potential benefits of autophagy modulators in human diseases.

## Chemical Screening Strategies for Identifying Autophagy Modulators

**FIGURE 2:**
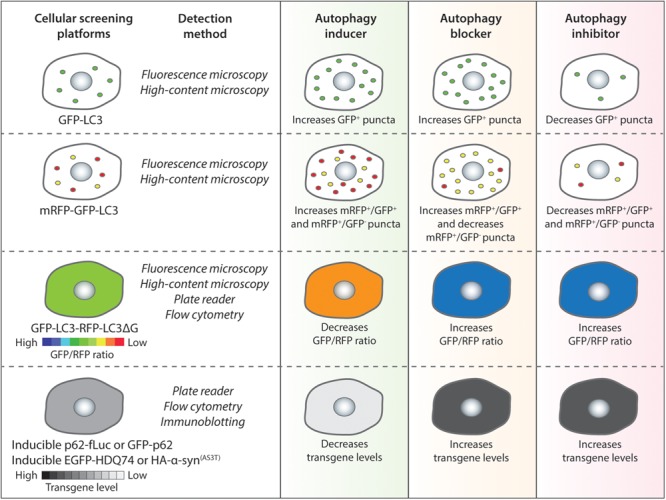
Autophagy chemical screening strategies in mammalian cells. Chemical screening methods that are commonly used for identifying autophagy modulators are based on autophagy reporters (LC3) or autophagy substrates (p62 or aggregation-prone proteins). The detection methods for the respective assays and the expected readouts for autophagy inducers, blockers or inhibitors are indicated as a general guidance.

A number of *in vitro* screening methods have been designed for identifying compounds (Sarkar, 2013a; Joachim et al., 2015; Seranova et al., 2019). The assays are primarily based on the perturbations of autophagy reporters or autophagy cargoes as readouts (Figure 1), which can be measured via fluorescence or high-content imaging, immunoblotting, flow cytometry and microplate reader (Mizushima et al., 2010; Klionsky et al., 2016; Figure 2 and Table 1). Some of these screening methods can be subjected to high-throughput applications. Below are descriptions of the common screening approaches in mammalian cells, and the identification and therapeutic benefits of key autophagy modulators.

**Table 1:** Chemical screening methods for identifying autophagy modulators in mammalian cells.

Autophagy screening assays	Detection methods	Strengths	Limitations
GFP-LC3	Fluorescence or high-content microscopy	(1) Simple readout easy to detect	(1) Can not distinguish between autophagy inducer and blocker
		(2) High-throughput application	(2) Can not assess overall autophagic flux
mRFP-EGFP-LC3	Fluorescence or high-content microscopy	(1) Can distinguish between autophagy inducer, inhibitor and blocker	(1) Assay depends on proper acidification of the lysosomes that can be affected by lysosomotrophic agents
		(2) Measures autophagosome flux	(2) Can not precisely assess overall autophagic flux as it does not measure cargo clearance.
		(3) High-throughput application	
GFP-LC3-RFP-LC3ΔG	Fluorescence or high-content microscopy, Flow cytometry, Microplate reader	(1) Measures overall autophagic flux	(1) Can not distinguish between autophagy inhibitor and blocker
		(2) Versatile detection methods	(2) Homologous recombination of two LC3 sequences could result in non-degradable GFP-LC3ΔG
		(3) High-throughput application	
Inducible p62-fLuc or GFP-p62	Microplate reader, Flow cytometry	(1) Measures clearance of autophagic cargo indicating overall autophagic flux	(1) Can not distinguish between autophagy inhibitor and blocker
		(2) Possible high-throughput application	(2) Transcriptional changes in leaky p62 transgene could affect readout
Inducible EGFP-HDQ74 or HA-α-syn^(A53T)^	Immunoblotting	(1) Measures clearance of autophagic cargo indicating overall autophagic flux	(1) Can not distinguish between autophagy inhibitor and blocker
			(2) High-throughput analysis not possible

*The detection methods, strengths and limitations of the autophagy reporter and substrate based screening assays are highlighted.*

## Chemical Screening Methods Based on Autophagy Reporters

Screening methods based on autophagy reporters are the most commonly used approaches to detect changes in the numbers of autophagosomes and autolysosomes (Table 1). The protein reporter that is widely used to study autophagy is microtubule-associated protein 1 (MAP1) light chain 3 (LC3). The nascent LC3 is cleaved at its C-terminal arginine residue by Atg4 to form the cytoplasmic LC3-I, which is then post-translationally conjugated with phosphatidylethanolamine at its C-terminal glycine residue by Atg7 to form the autophagosome-associated LC3-II (Kabeya et al., 2000). The lipidated LC3-II remains associated to the autophagosomes throughout their lifespan, and is present on both the outer and inner membranes. Following the maturation of autophagosomes with lysosomes to form autolysosomes, the LC3-II on the inner surface is degraded whereas the LC3-II on the outer surface is delipidated and removed by Atg4B for recycling (Tanida et al., 2004). A number of fluorescent-tagged reporters of LC3, such as GFP-LC3 (Kabeya et al., 2000), mRFP-GFP-LC3 (Kimura et al., 2007) and GFP-LC3-RFP-LC3ΔG (Kaizuka et al., 2016), have been used to study autophagy and undertake chemical screening.

### Identification of Autophagy Modulators by GFP-LC3 Screening Method

The most common LC3-based reporter that has been used in several studies is GFP-LC3, which labels autophagosomes, autolysosomes as well as phagophores (Kabeya et al., 2000). For the GFP-LC3 screening method, image-based analysis is done by quantifying the GFP^+^ puncta per cell to measure perturbations in autophagosome number. In general, an autophagy inducer as well as an autophagy blocker will increase GFP-LC3 puncta whereas an autophagy inhibitor will decrease GFP-LC3 puncta (Figure 2). A number of high-throughput and small-scale screens have been undertaken with this strategy that has been also utilized to assess the key hits arising from other screening methods; and some of the primary chemical screens utilizing GFP-LC3 readout are highlighted below.

Using GFP-LC3 as the primary screening method in a stable human glioblastoma H4 cell line, an image-based chemical screen with 480 bioactive compounds was performed wherein the number, size and intensity of GFP-LC3 spots were taken into consideration while selecting potent autophagy modulators (Zhang et al., 2007). Compounds were treated at 3–12 μM concentrations for 24 h. This screen identified 8 autophagy inducers, which included a number of FDA-approved drugs such as fluspirilene, trifluoperazine, pimozide (antipsychotic drugs), niguldipine, nicardipine, amiodarone (drugs used for cardiovascular conditions) and loperamide (used in diarrhea). While fluspirilene, trifluoperazine are dopamine antagonists, the other drugs are Ca^2+^ channel antagonists that lower intracellular Ca^2+^; all of which induced autophagy independently of mTOR (Zhang et al., 2007). Another image-based chemical screen was performed with a library of 3584 pharmacologically active compounds in human breast cancer MCF-7 cells stably expressing GFP-LC3 (Balgi et al., 2009). Treatment of compounds was done at ∼15 μM concentration for 4 h. This screen identified 3 FDA-approved drugs such as perhexilene, niclosamide and amiodarone, as well as rottlerin, as autophagy inducers; all of which were shown to inhibit mTORC1 (Balgi et al., 2009). However, other screens have reported amiodarone (Ca^2+^ channel antagonist) to act independently of mTORC1 for inducing autophagy at a much lower dose than what is required to inhibit mTORC1 (Williams et al., 2008); and likewise, perhexilene is a Ca^2+^ channel blocker that could be also mTOR-independent. Furthermore, one of the largest chemical screens for identifying autophagy modulators was undertaken in HeLa cells stably expressing GFP-LC3 with 59541 stereochemically and skeletally diverse compounds derived from diversity-oriented synthesis (Kuo et al., 2015). Compounds were treated for 4 h in 8-point dose with a maximal concentration of 10 μM. Several hits were subjected to a secondary screen at 10 μM concentration from which BRD5631 was identified as the potent autophagy inducer along with other hits like BRD2716 and BRD34009; all of which did not affect mTOR activity. Interestingly, the hit rate in the primary screen for compounds having an alkyl amine was higher than that for all of the compounds. This effect was augmented by the additional presence of a single lipophilic group, such as diphenyl alkyne, biphenyl, cyclohexane or naphthalene (Kuo et al., 2015). While the above screens were undertaken in immortalized human cell lines, another chemical screen was done with 1280 pharmacologically active compounds in mouse embryonic fibroblasts (MEFs) stably expressing GFP-LC3 (Li et al., 2016). Compounds were treated at 0.02–46 μM concentrations for 16 h in the presence or absence of chloroquine (autophagy blocker) to determine their effects on autophagic flux. Out of the 27 autophagy inducers identified, few were characterized further. These include anti-psychotic drugs such as indatraline hydrochloride (dopamine inhibitor), chlorpromazine hydrochloride and fluphenazine dihydrochloride (dopamine receptor antagonists). Fluphenazine was found to inhibit mTORC1 whereas indatraline and chlorpromazine were mTOR-independent (Li et al., 2016).

Although GFP-LC3 is a straightforward, widely-used screening assay, its inability to distinguish between autophagosomes and autolysosomes is a major inadequacy of this reporter. Accumulation of autophagosomes can occur either due to induction of autophagosome formation (by autophagy inducers) or due to block in autophagosome maturation (by autophagy blockers) in the early and late stages of autophagy, respectively (Rubinsztein et al., 2009). Since autophagy is a dynamic, multi-step process, it is imperative to measure autophagosome flux in order to assess the status of autophagy. Therefore, the hits from the primary GFP-LC3 screen are subjected to rigorous secondary assays (such as autophagosome formation and maturation, and autophagic substrate clearance, amongst others) (Mizushima et al., 2010; Klionsky et al., 2012) for characterizing autophagy modulators.

### Identification of Autophagy Modulators by mRFP-GFP-LC3 Screening Method

In order to overcome the problem of the GFP-LC3 reporter, a tandem fluorescent-tagged mRFP-GFP-LC3 reporter can be employed to determine autophagosome maturation for distinguishing between the autophagosomes and the autolysosomes. This mRFP-GFP-LC3 reporter is pH-sensitive. When overexpressed in cells, the autophagosomes exhibit both mRFP and GFP signals, whereas the autolysosomes emit only mRFP signal because the acid-labile GFP signal is quenched in the acidic environment (Kimura et al., 2007). For the mRFP-GFP-LC3 screening method, image-based analysis is done by quantifying the mRFP^+^ and GFP^+^ puncta per cell to measure perturbations in the number of autophagosomes (mRFP^+^/GFP^+^) and autolysosomes (mRFP^+^/GFP^-^). In general, an autophagy inducer (acting at early stage) will increase autophagosomes and autolysosomes, an autophagy inhibitor (acting at early stage) will decrease both these compartments, whereas an autophagy blocker (acting at late stage) will increase autophagosomes and decrease autolysosomes (Figure 2). Alternative versions of the mRFP-GFP-LC3 reporter have been described that may provide better readouts. These include replacing mRFP with mCherry that has superior photostability over mRFP (Pankiv et al., 2007), and substituting GFP with mWasabi that is more acid-sensitive than GFP (Zhou et al., 2012).

This pH-sensitive reporter has been primarily utilized as a secondary screening strategy following primary screens utilizing the more simpler GFP-LC3 method. In a high-throughput screen with 59541 compounds in GFP-LC3 platform, 400 screen hits were subjected to additional screening in stable HeLa cells expressing mCherry-GFP-LC3 (Kuo et al., 2015). These compounds were treated at 10 μM concentration for 24 h, after which 250 compounds increased (putative inducers) and 80 compounds decreased (putative inhibitors/blockers) the number of mCherry^+^/GFP^-^ autolysosomes. Following further characterization, potent mTOR-independent autophagy inducers identified were BRD5631, BRD2716, and BRD34009 (Kuo et al., 2015). In another study, HeLa cells stably expressing mRFP-GFP-LC3 was subjected to three drug libraries such as the Prestwick Chemical Library, Microsource Spectrum 2000 library and Johns Hopkins Library that encompass 3791 compounds including FDA-approved drugs and bioactive molecules (Chauhan et al., 2015). Compounds were treated at 10 μM concentration for 4 h. However, high-content image analysis was done based only on GFP-LC3 puncta and total integrated area per cell, but not together with mRFP-LC3 that was utilized later during secondary characterization. 80 compounds were identified, out of which 55 were novel and 25 were previously reported as autophagy modulators. Further characterization of the hits including the mRFP-GFP-LC3 analysis identified flubendazole as a novel autophagy inducer that is also an antihelminthic drug. Flubendazole was shown to impact on dynamic and acetylated microtubules to inhibit mTOR and disrupt Bcl2-Beclin 1 complex for inducing autophagy (Chauhan et al., 2015). More recently, a primary screen with mRFP-GFP-LC3 has been performed in U343 glioma cell spheroids (3D tumor spheroids) by dynamic live-cell imaging (Pampaloni et al., 2017). A subset of the Enzo Life Sciences Screen-Well Natural Compounds library comprising of 94 compounds were used at 1, 12.5, and 50 μM concentrations, followed by long-term time-lapse fluorescence imaging over 24 h at an interval of 1 h. Instead of measuring puncta formation, this study quantified the readout based on the ratio of mRFP and GFP emission intensities over time. Apart from validating this approach with the Enzo Life Sciences Screen-Well Autophagy library consisting of known autophagy modulators, the screen with selected natural compounds identified six potent autophagy inducers and four inhibitors. The autophagy-inducing natural compounds include PI-103, nonactin, valinomycin, quercetin, ivermectin, and harmine (Pampaloni et al., 2017).

The mRFP-GFP-LC3 reporter or its alternative versions can be subjected to high-throughput image-based screens to analyse autophagosome flux. This assay requires proper acidification of the lysosomes that could be affected by lysosomotrophic agents. However, autophagic substrate clearance along with other secondary assays should be assessed following the primary screen in order to assess the overall autophagic flux.

### Identification of Autophagy Modulators by GFP-LC3-RFP-LC3ΔG Screening Method

A novel autophagy probe, GFP-LC3-RFP-LC3ΔG, has been recently developed for evaluating autophagic flux that can be used for high-throughput screening approaches (Kaizuka et al., 2016). When overexpressed in cells, the Atg4 family proteases can cleave this reporter into equimolar amounts of GFP-LC3 and RFP-LC3ΔG. While GFP-LC3 on the autophagosomes is degraded or recycled after fusion with the lysosomes, RFP-LC3ΔG cannot be lipidated due to a deletion in its C-terminal glycine and thus remains in the cytosol serving as an internal control. This GFP-LC3-RFP-LC3ΔG reporter can be subjected to both qualitative (by ratiometric imaging via fluorescence microscopy) and quantitative (via microplate reader or flow cytometry) analyses by measuring the fluorescence of GFP-LC3 and RFP-LC3ΔG, and then calculating the GFP/RFP ratio (Kaizuka et al., 2016). Autophagy inducers are expected to decrease GFP/RFP ratio by enhancing autophagic flux, whereas autophagy inhibitors or blockers will increase GFP/RFP ratio by reducing autophagic flux (Figure 2).

Two chemical screens employing the GFP-LC3-RFP-LC3ΔG screening method have been undertaken using a selected library of 34 known autophagy-regulating compounds and 1054 approved drugs under basal or starvation conditions in HeLa cells stably expressing this reporter (Kaizuka et al., 2016). The GFP/RFP ratio was calculated from fluorescence measurement via a microplate reader. For the first screen with known autophagy-regulating compounds, cells were treated for 6, 12 or 24 h with concentrations previously shown to modulate autophagy. A number of known autophagy modulators, but not all, acted as expected primarily after 12 or 24 h treatment. Specifically, autophagy inducers such as rapamycin (Blommaart et al., 1995) and Torin 1 (Thoreen et al., 2009) decreased GFP/RFP ratio whereas autophagy blockers like bafilomycin A1 (Yamamoto et al., 1998) and chloroquine (Seglen et al., 1979) increased GFP/RFP ratio (Kaizuka et al., 2016). For the second screen with approved drug library, cells were treated for 24 h at 10 μM concentration with few exceptions at 5 μM. The screen hits included 47 autophagy-inducing drugs (comprising of certain anti-cancer drugs, antibiotics and cardiotonic drugs) and 43 autophagy inhibitory drugs. Although many of these hits were previously reported, 13 inducers and 18 inhibitors/blockers were identified as novel autophagy modulators, of which some of the novel autophagy inducers were adefovir pivoxil, methyltestosterone, norethisterone, oxaprozin, and zidovudine (Kaizuka et al., 2016). This GFP-LC3-RFP-LC3ΔG probe has been demonstrated to be capable of measuring basal and induced autophagic flux in Zebrafish and in tissues of transgenic mice (Kaizuka et al., 2016), and is thus valuable for monitoring autophagic flux *in vivo*.

Although this reporter can be used for high-throughput applications and *in vivo* studies to measure the overall autophagic flux, it is not ideal for investigating the distinct stages of autophagy such as autophagosome formation and maturation. Importantly, the two LC3 sequences of GFP-LC3-RFP-LC3ΔG in retrovirally transfected cells can undergo homologous recombination, which will generate GFP-LC3ΔG that is incapable of being degraded by autophagy. In addition, the expression levels of this reporter define the accuracy of the readout, and hence analysis in different cell lines or tissues will require comparable expression (Kaizuka et al., 2016; Geng and Klionsky, 2017).

## Chemical Screening Methods Based on Autophagy Substrates

In addition to the screening approaches based on LC3 reporters, autophagy substrate clearance has also been utilized as a primary screening assay for identifying autophagy modulators (Table 1). This method measures the autophagic cargo flux, which together with LC3-based secondary assays for autophagosome flux can indicate the overall autophagic flux.

### Identification of Autophagy Modulators by Clearance of Aggregation-Prone Proteins

A number of neurodegeneration-associated aggregation-prone proteins are predominantly degraded by autophagy (Menzies et al., 2017), and hence screening methods can be based on their clearance as readouts (Sarkar, 2013a). The well-established substrates undergoing autophagic degradation include mutant huntingtin (with expanded polyglutamine repeats) and mutant α-synuclein (A53T or A30P mutants) associated with Huntington’s and Parkinson’s disease, respectively (Webb et al., 2003; Ravikumar et al., 2004). Since the steady-state level of proteins is not ideal for accurately reflecting any impact on their degradation, stable inducible cell lines are required for analyzing autophagic substrate clearance where the transgene product is temporally synthesized by doxycycline followed by treatment with compounds after the expression is turned off (Wyttenbach et al., 2001; Webb et al., 2003; Sarkar et al., 2009). In general, autophagy inducers will enhance the clearance of aggregation-prone proteins, whereas autophagy inhibitors or blockers will retard their clearance (Figure 2).

Independent studies using a stable inducible PC12 cell line expressing EGFP-tagged mutant huntingtin (EGFP-HDQ74) identified mTOR-independent autophagy inducers such as trehalose (Sarkar et al., 2007a) as well as inositol-lowering agents (lithium, carbamazepine, valproic acid, L-690330) (Sarkar et al., 2005) and nitric oxide synthase inhibitors (L-NAME) (Sarkar et al., 2011). These studies also identified autophagy inhibitory compounds such as agents increasing inositol or inositol 1,4,5-trisphosphate (IP_3_) levels (myo-inositol, prolyl endopeptidase inhibitor 2) (Sarkar et al., 2005) and nitric oxide donors (DEA NONOate, DETA NONOate) (Sarkar et al., 2011). Utilizing stable inducible PC12 cell line expressing hemagglutinin (HA)-tagged A53T α-synuclein (HA-α-syn^(A53T)^) as the primary screening method, a chemical screen was undertaken with 72 hits arising from an yeast screen involving 50729 compounds (Sarkar et al., 2007b). Cells were treated with compounds at 2 mg mL^-1^ concentration for 24 h after the initial doxycycline-induced synthesis of the transgene product (A53T α-synuclein), followed by immunoblotting analysis to measure its clearance. A number of novel autophagy modulators were identified which enhanced the autophagy substrate clearance. These include 4 small molecule enhancers of rapamycin (SMERs) and 13 small molecule inhibitors of rapamycin (SMIRs), of which SMER10, SMER18, and SMER28 were characterized to be autophagy inducers acting independently of mTOR. Further screening of the chemical analogs of these SMERs identified 18 additional autophagy inducers, such as 1 SMER10, 7 SMER18 and 10 SMER28 analogs that are capable of enhancing substrate clearance; although not substantially better than the respective parent compounds (Sarkar et al., 2007b). Another screen also utilizing a stable inducible PC12 cell line expressing HA-tagged A30P α-synuclein (HA-α-syn^(A30P)^) was undertaken with a library of 253 compounds including FDA-approved drugs and pharmacological probes (Williams et al., 2008). Drug treatment was done at 1 μM for 24 h after the synthesis of the transgene product, followed by immunoblotting analysis. This study elucidated a cyclic mTOR-independent autophagy pathway with multiple drug targets, in which cAMP regulates IP_3_ levels that impact on calpain activity, which in turn activates G_sα_ that regulates cAMP levels. Some of the autophagy-inducing compounds identified include L-type Ca^2+^ channel blockers (verapamil, loperamide, amiodarone), calpain inhibitors (calpastatin), ATP-sensitive K^+^ channel agonist (minoxidil), cAMP reducing agents (rilmenidine, clonidine) and inositol lowering agents (valproic acid), whereas Ca^2+^ channel openers [(±)-Bay K8644] and agents elevating cAMP (dibutyryl cAMP, forskolin) and cytosolic Ca^2+^ (thapsigargin) levels were autophagy inhibitory (Williams et al., 2008). In addition to these immunoblotting based methods, the effects of autophagy modulators on autophagy-dependent clearance of EGFP-tagged mutant huntingtin aggregates can be validated by fluorescence microscopy in wild-type (*Atg5^+/+^*) and autophagy-deficient (*Atg5*^-^*^/^*^-^) mouse embryonic fibroblasts (MEFs) (Kuma et al., 2004; Sarkar et al., 2009).

Although autophagic clearance of aggregation-prone proteins is informative for autophagic flux, only low-throughput approaches are possible that creates a major hurdle for high-throughput applications. Nonetheless, this method could be used as a secondary assay for characterization of selected hits arising from screens with LC3-based reporters.

### Identification of Autophagy Modulators by p62/SQSTM1 Clearance

An alternative approach to the clearance of aggregation-prone proteins is to monitor the autophagic degradation of a known autophagy substrate, p62/SQSTM1, which also functions as an adaptor protein during selective autophagy for recruiting specific autophagic cargo to the autophagosomes (Bjorkoy et al., 2005; Pankiv et al., 2007). Similarly, to the method involving aggregation-prone proteins, screening approaches based on p62 clearance would ideally require a stable inducible cell line where the transgene product is temporally expressed before the treatment with compounds. The p62 reporters, such as GFP-p62 (Larsen et al., 2010) or luciferase-tagged p62 (Brown et al., 2016; Min et al., 2018), could be utilized for medium- to high-throughput screens by flow cytometry or microplate reader (for analyzing p62 levels) or by fluorescence imaging (for analyzing p62 aggregates). Genetic screens have been undertaken with p62-based reporters (Pietrocola et al., 2015; Strohecker et al., 2015; DeJesus et al., 2016; Hale et al., 2016), and therefore, similar chemical screening approaches are also possible. In addition, analyzing the steady-state levels of endogenous p62 by immunoblotting is often used as a secondary assay for characterization of autophagy modulators (Klionsky et al., 2012). It is expected that an autophagy inducer will decrease p62 levels or aggregates, whereas an autophagy inhibitor or blocker will cause its accumulation (Figure 2). Recently, an assay based on LC3B-II and p62 time-resolved fluorescence resonance energy transfer (TR-FRET) has been described to monitor autophagy independent of any exogenous labels. This method is based on the proximity of the donor and the acceptor antibodies of LC3-II and p62, in which autophagy inducers increase LC3-II signal and decrease p62 signal, autophagy inhibitors do not display any turnover of either signals, whereas autophagy blockers will increase LC3-II signal without any turnover of p62 signal (Bresciani et al., 2018).

Although p62 is a specific autophagy substrate in most mammalian cell lines (Klionsky et al., 2012), its autophagic degradation should be confirmed in the cell-type and the time-points to be used in the screens. Moreover, transcriptional upregulation of p62 has been reported during some instances of autophagy activation, such as under prolonged starvation or with certain pharmacological inducers (Klionsky et al., 2012; Sahani et al., 2014; Kuo et al., 2015), and therefore, any perturbation in p62 protein levels needs to be accompanied by qPCR assessment of its mRNA levels.

## Biomedical Applications of Autophagy Modulators in Human Diseases

**FIGURE 3:**
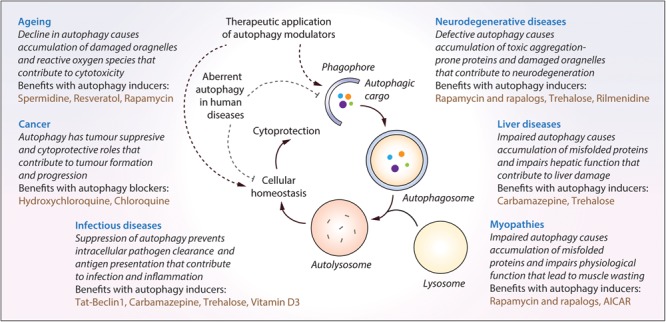
The impact of malfunctioning autophagy and the therapeutic benefits of autophagy modulators in diverse human diseases. Autophagy is implicated in diverse human diseases due to its vital role in maintaining cellular homeostasis. Defective or aberrant autophagy contributes to the cytotoxicity underlying many pathological conditions whereas pharmacological upregulation of autophagy is beneficial in various transgenic models. Key autophagy modulators exerting therapeutic benefits in neurodegenerative disorders, cancer, infectious diseases, liver diseases, myopathies and lifespan extension, as well as the impact of malfunctioning autophagy in these contexts, are highlighted.

Autophagy plays an essential role for tissue homeostasis and cellular survival by removing unwanted materials like malfunctioning aggregated proteins and damaged organelles from the cells; however, deregulation of this process could contribute to cytotoxicity (Mizushima et al., 2008). Autophagy dysfunction has been implicated in the pathogenesis of diverse human diseases (Levine and Kroemer, 2008; Jiang and Mizushima, 2014), and therefore, therapeutic exploitation of autophagy is of potential biomedical relevance (Figure 3). A number of independent studies and chemical screens have identified several autophagy modulators, which have been shown to impart beneficial effects in various transgenic disease models (Table 2; Rubinsztein et al., 2012; Sarkar, 2013b; Levine et al., 2015). Some of the key studies in specific disease contexts are highlighted below.

**Table 2:** Therapeutic benefits of autophagy modulators in diverse human diseases.

Diseases	Selected autophagy modulators	Mechanisms of autophagy modulation	Therapeutic benefits in animal and iPSC models
Neurodegenerative diseases	Rapamycin, CCI-779 (Inducers)	Inhibition of mTORC1 (Blommaart et al., 1995; Ravikumar et al., 2004)	HD flies (Ravikumar et al., 2004; Sarkar et al., 2008), FTD flies (Berger et al., 2006), HD mice (Ravikumar et al., 2004), AD mice (Spilman et al., 2010), FTD mice (Wang et al., 2012; Ozcelik et al., 2013; Jiang et al., 2014), SCA3 mice (Menzies et al., 2010), Prion disease mice (Cortes et al., 2012)
	Lithium (Inducer)	Reduction of inositol and IP_3_; mTORC1-independent (Sarkar et al., 2005)	HD flies (Sarkar et al., 2008), AD mice (Zhang et al., 2011), FTD mice (Shimada et al., 2012), ALS mice (Fornai et al., 2008)
	Carbamazepine (Inducer)	Reduction of inositol and IP_3_; mTORC1-independent (Sarkar et al., 2005)	AD mice (Li et al., 2013), FTD mice (Wang et al., 2012), ALS mice (Zhang et al., 2018), NPC1 patient iPSC-derived neurons (Maetzel et al., 2014)
	Trehalose (Inducer)	mTORC1-independent (Sarkar et al., 2007a); Inhibition of SLC2A and activation of AMPK (DeBosch et al., 2016)	HD mice (Tanaka et al., 2004), AD mice (Du et al., 2013), PD mice (Tanji et al., 2015), FTD mice (Rodriguez-Navarro et al., 2010; Schaeffer et al., 2012), SCA17 mice (Chen et al., 2015), ALS mice (Castillo et al., 2013; Zhang et al., 2014), NPC1 patient iPSC-derived neurons (Maetzel et al., 2014)
	Rilmenidine, Clonidine (Inducers)	Reduction of cAMP; mTORC1 independent (Williams et al., 2008)	HD mice (Rose et al., 2010), HD zebrafish (Williams et al., 2008), HD flies (Williams et al., 2008)
	Verapamil (Inducer)	Reduction of Ca^2+^; mTORC1 independent (Williams et al., 2008)	HD zebrafish (Williams et al., 2008), HD flies (Williams et al., 2008), NPC1 patient iPSC-derived neurons (Maetzel et al., 2014)
	SMER28 (Inducer)	Mechanism unknown; mTORC1 independent (Sarkar et al., 2007b)	HD flies (Sarkar et al., 2007b)
	BRD5631 (Inducer)	Mechanism unknown; mTORC1 independent (Kuo et al., 2015)	NPC1 patient iPSC-derived neurons (Kuo et al., 2015)
	Metformin (Inducer)	Activation of AMPK (Buzzai et al., 2007)	HD mice (Ma et al., 2007), LD mice (Berthier et al., 2016)
	6-Bio (Inducer)	Inhibition of mTORC1 signaling (Suresh et al., 2017)	PD mice (Suresh et al., 2017)
	AUTEN-67, AUTEN-99 (Inducers)	Inhibition of MTMR14 (Papp et al., 2016; Kovacs et al., 2017)	HD flies (Billes et al., 2016; Papp et al., 2016; Kovacs et al., 2017), PD flies (Kovacs et al., 2017)
Cancer	Chloroquine, Hydroxychloroquine (Blockers)	Mechanism unknown; Impairment of lysosomal acidification and autophagosome-lysosome fusion (Murakami et al., 1998; Boya et al., 2005)	*Myc/p53ER^TAM^* induced lymphoma mice (Amaravadi et al., 2007), mice bearing MCF7-RR and LCC9 ER+ breast cancer xenografts (Cook et al., 2014)
	Lys05, ROC-325 (Blockers)	Mechanism unknown; Impairment of lysosomal acidification and autophagosome-lysosome fusion (McAfee et al., 2012; Carew et al., 2017)	Mice bearing c8161 melanoma, 1205Lu melanoma and HT-29 colon cancer xenografts (McAfee et al., 2012), mice bearing 786-0 RCC xenografts (Carew et al., 2017)
	NSC185058, UAMC-2526 (Inhibitors)	Inhibition of ATG4B (Akin et al., 2014; Kurdi et al., 2017)	Mice bearing Saos-2 osteosarcoma xenograft (Akin et al., 2014), Mice bearing HT29 colorectal tumor xenograft (Kurdi et al., 2017)
	Pyrvinium pamoate (Inhibitor)	Mechanism unknown; Reduction in *Atg* gene expression; mTORC1 independent (Deng et al., 2013)	Mice bearing 4TI mammary carcinoma xenograft (Deng et al., 2013)
	Torin 1 (Inducer)	ATP-competitive inhibition of mTORC1 (Thoreen et al., 2009)	Mice bearing Tu12 and Tu22 colon cancer xenografts (Francipane and Lagasse, 2013)
Infectious diseases	Tat-Beclin 1 (Inducer)	Interaction with the negative autophagy regulator GAPR-1 (Shoji-Kawata et al., 2013)	Mice infected with chikungunya or West Nile virus (Shoji-Kawata et al., 2013), murine or human macrophages infected with *L. monocytogenes* bacteria and HIV (Shoji-Kawata et al., 2013)
	Vitamin D3 (Inducer)	Increase in Beclin 1 (Wang et al., 2008); Increase in *Atg* gene expression (Yuk et al., 2009)	Human macrophages infected with *M. tuberculosis bacteria* or HIV or coinfection (Yuk et al., 2009; Campbell and Spector, 2011, 2012)
	Carbamazepine (Inducer)	Reduction of inositol and IP_3_; mTORC1-independent (Sarkar et al., 2005)	Human macrophages infected with *M. tuberculosis bacteria* or coinfection with HIV (Schiebler et al., 2015), mice infected with multidrug-resistant *M. tuberculosis bacteria* (Schiebler et al., 2015)
	Trehalose (Inducer)	mTORC1-independent (Sarkar et al., 2007a); PI(3,5)P_2_ agonist, activation of TRPML1 Ca^2+^ channel (Sharma et al., 2017)	Human macrophages infected with *M. tuberculosis bacteria* or coinfection with HIV (Sharma et al., 2017), PBMCs from HIV patients (Sharma et al., 2017)
	Flubendazole (Inducer)	mTORC1 inactivation; nuclear translocation of TFEB (Chauhan et al., 2015)	Human dendritic cells infected with *HIV*, and HeLa cells infected with *E. coli bacteria* (Chauhan et al., 2015)
	Nitazoxanide (Inducer)	Inhibition of mTORC1 signaling (Lam et al., 2012)	Human acute monocytic leukemia cells or PBMCs infected with *M. tuberculosis bacteria* (Lam et al., 2012)
	Nortriptyline (Inducer)	Mechanism unknown	Human macrophages infected with *M. tuberculosis bacteria* (Sundaramurthy et al., 2013)
Liver Disease	Carbamazepine (Inducer)	Reduction of inositol and IP_3_; mTORC1-independent (Sarkar et al., 2005)	AATD mice (Hidvegi et al., 2010), NAFLD and AFLD mice (Lin et al., 2013), FSD patients (Puls et al., 2013), AATD patient iPSC-derived hepatic cells (Choi et al., 2013), NPC1 patient iPSC-derived hepatic cells (Maetzel et al., 2014)
	Lithium, Valproic acid (Inducers)	Reduction of inositol and IP_3_; mTORC1-independent (Sarkar et al., 2005)	AATD patient iPSC-derived hepatic cells (Choi et al., 2013)
	Trehalose (Inducer)	mTORC1-independent (Sarkar et al., 2007a); Inhibition of SLC2A and activation of AMPK (DeBosch et al., 2016)	NAFLD mice (DeBosch et al., 2016)
	Rapamycin (Inducer)	Inhibition of mTORC1 (Blommaart et al., 1995)	NAFLD mice (Lin et al., 2013), NPC1 patient iPSC-derived hepatic cells (Maetzel et al., 2014)
Myopathies	Rapamycin, CCI-779 (Inducers)	Inhibition of mTORC1 (Blommaart et al., 1995; Ravikumar et al., 2004)	Collagen type VI muscular dystrophy mice (Grumati et al., 2010), *LMNA* cardiomyopathy mice (Choi et al., 2012; Ramos et al., 2012)
	AICAR (Inducer)	Activation of AMPK (Buzzai et al., 2007)	DMD mice (Pauly et al., 2012)
	Simvastatin (Inducer)	Inhibition of Rac1-mTOR pathway (Wei et al., 2013)	DMD mice (Whitehead et al., 2015)
Lifespan extension	Spermidine (Inducer)	Inhibition of histone acetyltransferase and increase in *Atg* gene expression (Eisenberg et al., 2009)	Flies (Eisenberg et al., 2009), worms (Eisenberg et al., 2009), mice (Eisenberg et al., 2016)
	Resveratrol (Inducer)	Activation of SIRT1 (Morselli et al., 2010)	Flies (Wood et al., 2004), worms (Wood et al., 2004; Morselli et al., 2010), mice (Baur et al., 2006)
	Rapamycin (Inducer)	Inhibition of mTORC1 (Blommaart et al., 1995)	Flies (Bjedov et al., 2010), mice (Harrison et al., 2009)

*Autophagy modulators have shown beneficial effects in a number of transgenic disease models, such as but not limited to, neurodegenerative disorders, cancer, infectious diseases, liver diseases and myopathies as well as in lifespan extension. Selected examples of autophagy modulators are highlighted in specific pathological contexts. AATD, α1 antitrypsin deficiency; AD, Alzheimer’s disease; AFLD, Alcoholic fatty liver disease; ALS, Amyotrophic lateral sclerosis; AMPK, 5′ adenosine monophosphate-activated protein kinase; *Atg*, Autophagy-related genes; cAMP, 3′,5′-cyclic adenosine monophosphate; DMD, Duchenne muscular dystrophy; FSD, Fibrinogen storage disease, FTD, Frontotemporal dementia; GAPR-1, Golgi-associated plant pathogenesis-related protein 1; HD, Huntington’s disease; HIV, Human immunodeficiency virus; IP_*3*_, Inositol 1,4,5-trisphosphate; iPSC, Induced pluripotent stem cells; LD, Lafora disease; *LMNA*, Lamin A/C gene; MTMR14, Myotubularin related protein 14; mTORC1, Mechanistic target of rapamycin complex 1; NAFLD, Non-alcoholic fatty liver disease; NPC1, Niemann-Pick type C1 disease; PBMC, Peripheral blood mononuclear cells; PD, Parkinson’s disease; PI(3,5)P_*2*_, Phosphatidylinositol *3,5*-bisphosphate; RCC, Renal cell carcinoma; SCA, Spinocerebellar ataxia; SIRT1 Sirtuin 1; SLC2A, Solute carrier 2A; TRPML1, Transient receptor potential cation channel mucolipin subfamily member 1.*

## Autophagy Modulators in Neurodegenerative Diseases

Basal autophagy in the brain is critical for maintaining cellular homeostasis in post-mitotic cells like neurons, which is evident from the genetic studies in mice where brain-specific deletion of essential autophagy genes resulted in neurodegenerative phenotypes (Hara et al., 2006; Komatsu et al., 2006). Particularly, autophagy is the primary degradation pathway for several aggregation-prone proteins associated with neurodegeneration (Rubinsztein, 2006; Nixon, 2013). However, defective autophagy has been reported in several neurodegenerative diseases, including neurodegenerative lysosomal storage disorders, and is considered a major causative factor for neurodegeneration (Nixon, 2013; Sarkar, 2013b; Menzies et al., 2017; Seranova et al., 2017). Therefore, induction of autophagy for enhancing the clearance of mutant aggregation-prone proteins is considered a potential treatment strategy. The therapeutic benefits of autophagy inducers have been robustly demonstrated in the context of neurodegeneration where upregulation of autophagy was protective in several *in vitro* and *in vivo* transgenic models of neurodegenerative diseases (Rubinsztein et al., 2012; Sarkar, 2013b; Levine et al., 2015; Seranova et al., 2017). Stimulating autophagy with mTOR inhibitors like rapamycin or its analogs had beneficial effects in fly and mouse models of Huntington’s disease, Alzheimer’s disease (AD), Parkinson’s disease (PD), frontotemporal dementia (FTD), spinocerebellar ataxia type 3 (SCA3) and prion disease (Ravikumar et al., 2004; Berger et al., 2006; Sarkar et al., 2008; Menzies et al., 2010; Spilman et al., 2010; Cortes et al., 2012; Wang et al., 2012; Ozcelik et al., 2013; Jiang et al., 2014). Likewise, several mTOR-independent autophagy inducers such as, but not limited to, lithium, carbamazepine (inositol lowering agents), rilmenidine (cAMP reducing agent), trehalose (AMPK activator), SMERs and BRD5631 have been shown to be protective in fly, Zebrafish, mouse or induced pluripotent stem cell (iPSC) models of AD, FTD, HD, amyotrophic lateral sclerosis (ALS) and Niemann-Pick type C1 (NPC1) disease (Sarkar et al., 2005, 2007a,b; Fornai et al., 2008; Williams et al., 2008; Rose et al., 2010; Zhang et al., 2011, 2018; Shimada et al., 2012; Wang et al., 2012; Li et al., 2013; Maetzel et al., 2014; Kuo et al., 2015). The most widely used mTOR-independent autophagy inducer *in vivo* is trehalose (Sarkar et al., 2007a), a disaccharide that stimulates autophagy by inhibiting SLC2A family of glucose transporters and activating AMPK (DeBosch et al., 2016), which in turn can directly influence the phosphorylation of the autophagy-initiating kinase ULK1 (Egan et al., 2011; Kim et al., 2011). Remarkably, trehalose had beneficial effects in mouse models of AD, PD, HD, FTD, SCA17, ALS, as well as cellular and iPSC-derived neuronal models of prion and NPC1 disease, respectively (Tanaka et al., 2004; Aguib et al., 2009; Rodriguez-Navarro et al., 2010; Schaeffer et al., 2012; Castillo et al., 2013; Du et al., 2013; Zhang et al., 2014; Chen et al., 2015; Tanji et al., 2015). Additional autophagy-inducing agents reported to be cytoprotective in neurodegenerative models such as HD, PD, ALS, FTD and Lafora disease include Tat-Beclin 1 peptide, calpastatin, verapamil, metformin, AUTEN-67, AUTEN-99, 6-Bio and fluphenazine (Ma et al., 2007; Williams et al., 2008; Shoji-Kawata et al., 2013; Barmada et al., 2014; Berthier et al., 2016; Billes et al., 2016; Papp et al., 2016; Kovacs et al., 2017; Suresh et al., 2017). A combinatorial approach in enhancing autophagy has been shown with rapamycin and mTOR-independent autophagy inducers such as lithium, trehalose or SMERs. Higher efficacy was achieved via the additive effects of dual treatment on autophagy induction and cytoprotection in cell and fly models of HD than the effects of single compounds (Sarkar et al., 2007a,b, 2008).

## Autophagy Modulators in Cancer

The ability of autophagy in the maintenance of metabolic homeostasis has drawn considerable attention as a potential target for cancer therapy via its pro-survival and pro-death mechanisms (Rabinowitz and White, 2010; Levy et al., 2017). Autophagy plays tumor suppressive role by mitigating oxidative stress, removing superfluous mitochondria and preventing DNA damage and genome instability; and on the other hand, shows pro-tumor activity by preventing the induction of tumor suppressors, increasing resistance to apoptosis and maintaining tumor metabolism through recycling of nutrients (Mathew et al., 2007; Galluzzi et al., 2015; Kimmelman and White, 2017). Depending on the cancer context and the opposing effects of autophagy, either inhibitors or inducers of autophagy could be exploited for cancer therapy (Galluzzi et al., 2017; Levy et al., 2017). Since autophagy promotes tumorigenesis in most contexts, inhibition of autophagy has gathered considerable interest for cancer therapy. Accumulating evidence demonstrate that autophagy inhibitors/blockers exerted therapeutic benefits in cancer models. The clinically- approved autophagy inhibitors chloroquine or hydroxychloroquine (HCQ), which impair lysosomal acidification and block autophagic flux (Murakami et al., 1998; Boya et al., 2005), caused tumor shrinkage in preclinical studies; and thus HCQ being more potent with lesser side-effects is used in ongoing clinical trials either alone or in combination with other treatments (Briceno et al., 2003; Amaravadi et al., 2007; Cook et al., 2014; Chude and Amaravadi, 2017; Levy et al., 2017; Onorati et al., 2018). Autophagy inhibitory compounds, such as Lys05 and ROC-325, which exhibited anti-tumor activity in mice have been suggested to be more potent than HCQ (McAfee et al., 2012; Carew et al., 2017). In addition, autophagy inhibitors preventing autophagosome formation such as ATG4B antagonists (compounds NSC185058 and UAMC-2526), Vps34 (vacuolar protein sorting protein 34) inhibitor (compound SAR405), ULK1 (Unc-51-like kinase 1) inhibitor (compound SBI-0206965), USP10/USP13 (ubiquitin-specific peptidases) inhibitor (Spautin-1) and agents causing transcriptional inhibition of autophagy genes (pyrvinium pamoate), also exerted anti-proliferative and anti-tumor effects in cellular and *in vivo* models of cancer (Liu et al., 2011; Deng et al., 2013; Akin et al., 2014; Ronan et al., 2014; Shao et al., 2014; Egan et al., 2015; Kurdi et al., 2017). On the contrary, various chemical agents or natural products exerting antiproliferative or anti-tumor activity either alone or in combination with chemotherapeutic agents could induce autophagy or autophagic cell death, which include Torin 1, AC-73, MC-4, metformin, silibinin, Abrus agglutinin, curcumin, liensinine, spermidine, vitamin D3, and imatinib (Buzzai et al., 2007; Ertmer et al., 2007; Wang et al., 2008; Thoreen et al., 2009; Qian et al., 2011; Francipane and Lagasse, 2013; Law et al., 2014; Jiang et al., 2016; Pietrocola et al., 2016; Panda et al., 2017; Son et al., 2018; Spinello et al., 2018).

## Autophagy Modulators in Infectious Diseases

Autophagy plays an important role in innate defense mechanism by removing intracellular pathogens; a process termed xenophagy (Levine et al., 2011; Deretic et al., 2013). The role of autophagy in regulating intracellular infections initially emerged through studies on *Mycobacterium tuberculosis* (*Mtb*) (Gutierrez et al., 2004; Singh et al., 2006). Subsequently, several other bacterial pathogens like *Salmonella* and *Listeria*, and viral pathogens like HIV and Dengue were shown to utilize host autophagy pathways for their own advantage (Jia et al., 2009; Kyei et al., 2009; Yoshikawa et al., 2009; Heaton and Randall, 2010). A genome-wide siRNA screen to identify host factors required for intracellular *Mtb* survival within macrophages revealed that a large number of host factors acted via regulation of autophagy to help the bacteria (Kumar et al., 2010). Induction of autophagy with rapamycin, carbamazepine, SMER28, and vitamin D3 were shown to prevent bacterial survival or HIV replication in macrophages (Gutierrez et al., 2004; Floto et al., 2007; Yuk et al., 2009; Kumar et al., 2010; Campbell and Spector, 2011, 2012; Schiebler et al., 2015). Notably, carbamazepine reduced bacterial burden, improved lung pathology and stimulated adaptive immunity in mice infected with multidrug-resistant *Mtb* (Schiebler et al., 2015). Rapamycin also controlled viral and bacterial pathogens both *in vitro* and *in vivo* (Donia et al., 2010). In an integrated chemical and RNAi screening for modulators of intracellular mycobacteria, one of the top three compounds was nortriptyline which significantly suppressed *Mtb* survival within macrophages and induced autophagy (Sundaramurthy et al., 2013). Other compounds limiting bacterial or HIV infections through activation of autophagic flux were nitazoxanide (anti-protozoan drug) and flubendazole (antihelminthic drug) (Lam et al., 2012; Chauhan et al., 2015). Similarly, the naturally occurring disaccharide trehalose, a potent mTOR-independent enhancer of autophagy in diverse cell-types (Sarkar et al., 2007a), can also induce autophagy and xenophagy in *Mtb*-infected macrophages that resulted in the killing of bacteria (Sharma et al., 2017). In this study, trehalose was found to act as a PI(3,5)P_2_ (phosphatidylinositol *3,5*-bisphosphate) agonist for activating the lysosomal Ca^2+^ channel TRPML1 (Sharma et al., 2017), which in turn released lysosomal Ca^2+^ that caused nuclear translocation of TFEB to induce autophagy (Medina et al., 2015). Excitingly, trehalose also seemed to be effective during HIV-*Mtb* co-infection and limits *Mtb* survival by reversing the HIV-mediated block in autophagy flux (Sharma et al., 2017). Similarly, vitamin D3 could also kill *Mtb* during HIV co-infection by inducing autophagy (Campbell and Spector, 2012). Several host factors currently being tested for anti-*Mtb* therapeutics function by regulating host autophagy and xenophagy. For example, inhibition of host Src kinase by the compound AZD0530 induced autophagy and lysosomal maturation to clear *Mtb* (Chandra et al., 2016). A pioneering anti-infective, autophagy-inducing agent is Tat-Beclin 1, which is a peptide representing a region of the autophagy regulator Beclin 1 that interacts with the HIV-1 accessory protein NEF, and this domain is linked with the HIV-1 Tat transduction domain to make it cell permeable (Shoji-Kawata et al., 2013). Tat-Beclin 1 prevented the replication of a number of viral and bacterial pathogens *in vitro* in autophagy-dependent manner, as well as induced autophagy and anti-viral activity in mice infected with chikungunya or West Nile virus (Shoji-Kawata et al., 2013). Thus, it is evident that regulators of autophagy and xenophagy have tremendous potential for novel therapeutics against various infectious diseases. It is now clear that within an infected host cell, there is a possibility of uncoupling between homeostatic autophagy and anti-bacterial xenophagy (Chandra et al., 2015; Sharma et al., 2018). Therefore, it is desirable to perform chemical screening pertaining to infection-specific xenophagy flux for identifying novel regulators of bacterial/viral survival within the host cells through the autophagy pathway.

## Autophagy Modulators in Liver Diseases

Liver autophagy is essential for various hepatic functions and is implicated in various liver conditions including α1-antitrypsin (AAT) deficiency, non-alcoholic fatty liver disease (NAFLD), hepatocellular carcinoma and viral hepatitis (Rautou et al., 2010; Ueno and Komatsu, 2017). Chemical modulation of autophagy has been shown to have beneficial effects in some of these diseases. Carbamazepine, an mTOR independent autophagy inducer acting by reducing inositol levels (Sarkar et al., 2005), reduced hepatic load of mutant α1-antitrypsin Z and hepatic fibrosis in a mouse model of AAT deficiency (Hidvegi et al., 2010), as well as decreased hepatocellular aggregate-related toxicity in patients suffering from fibrinogen storage disease (Puls et al., 2013). A high-throughput drug screen in hepatocyte-like cells derived from iPSC lines of patients with AAT deficiency also revealed inositol-lowering autophagy-inducing agents, such as carbamazepine, lithium, and valproic acid, in facilitating the clearance mutant AAT (Choi et al., 2013). Carbamazepine as well as the mTOR inhibitor rapamycin also rescued dysfunctional autophagic flux and improved cell viability in hepatic-like cells differentiated from patient-derived iPSC lines of Niemann-Pick type C1 (NPC1) disease (Maetzel et al., 2014). In addition, autophagy induction with trehalose, carbamazepine, rapamycin or hydrogen sulfide reduced steatosis, lipid accumulation and liver injury in high-fat diet-induced NAFLD in mice (Lin et al., 2013; Sun et al., 2015; DeBosch et al., 2016). Furthermore, the anti-diabetic drug metformin, which indirectly inhibits mTOR, induced SIRT1-mediated autophagy in primary hepatocytes and ameliorated hepatic steatosis *in vivo* (Song et al., 2015). Overall, these studies indicate that activation of autophagy via inhibition of mTOR, lowering inositol levels or with trehalose are effective modes of inducing autophagy in the liver.

## Autophagy Modulators in Myopathies

Basal autophagy is required for maintaining muscle mass and myofiber integrity (Masiero et al., 2009), and thus deregulation of muscle autophagy is implicated in myopathies and muscular dystrophies (Sandri et al., 2013). Sustained activation of mTORC1 in skeletal muscle of TSC1-deficient mice could cause late-onset myopathy related to suppression of autophagy (Castets et al., 2013). Upregulation of autophagy, primarily by inhibiting the mTORC1 pathway, has been reported to have beneficial effects in certain transgenic disease models. Autophagy induction by rapamycin or low-protein diet increased myofiber survival and attenuated dystrophic phenotype in a mouse model of collagen type VI muscular dystrophy (Grumati et al., 2010). Likewise, activation of autophagy by dietary changes or with the AMP-activated protein kinase (AMPK) agonist, AICAR (5-aminoimidazole-4-carboxamide-1-β-d-ribofuranoside), improved dystrophic phenotypes in mouse models of Duchenne muscular dystrophy (DMD) (De Palma et al., 2012; Pauly et al., 2012). A potential role of simvastatin, which has been reported to induce autophagy by inhibiting the Rac1-mTOR pathway (Wei et al., 2013), has been suggested in improving the physiological function of skeletal muscle in DMD transgenic mice (Whitehead et al., 2015). In addition, rapamycin or its analog, temsirolimus, ameliorated cardiomyopathy and improved skeletal and cardiac muscle function in mouse models of *LMNA* (lamin A/C gene) cardiomyopathy that recapitulate Emery-Dreifuss muscular dystrophy (EDMD) (Choi et al., 2012; Ramos et al., 2012).

## Autophagy Modulators in Lifespan Extension

The functionality of autophagy declines with aging (Rubinsztein et al., 2011), and thus restoring adequate autophagy is considered a possible anti-aging strategy for lifespan extension. There are a number of lifespan expanding strategies, and in many of such approaches, autophagy acts as a common denominator for promoting longevity (Madeo et al., 2010; Hansen et al., 2018). Pharmacological treatment with autophagy inducers has been linked to increasing longevity in transgenic *in vivo* models (Madeo et al., 2015). Lifespan extension via induction of autophagy with naturally- occurring polyamines such as spermidine, which is an acetyltransferase inhibitor, was shown in yeast, flies, worms and mice (Eisenberg et al., 2009, 2016); and likewise also reported with the natural phenol resveratrol, which is a deacetylase activator, in yeast, flies, worms as well as in mice on high-fat diet (Howitz et al., 2003; Wood et al., 2004; Baur et al., 2006; Morselli et al., 2010). Although both spermidine and resveratrol impacts on the acetylproteome, stimulation of autophagy by resveratrol requires the nicotinamide adenine dinucleotide-dependent deacetylase sirtuin 1 (SIRT1) whereas the effect of spermidine was SIRT1 independent (Morselli et al., 2010, 2011). Inhibition of mTOR by rapamycin also extended lifespan in yeast, flies and mice (Alvers et al., 2009; Harrison et al., 2009; Bjedov et al., 2010; Lamming et al., 2013). In addition, lifespan extension in multiple organisms including mice and apes could be achieved by caloric restriction, which is a physiological inducer of autophagy via AMPK activation, mTORC1 inhibition and SIRT1 activation (Mair and Dillin, 2008; Colman et al., 2009; Mercken et al., 2014; Mattison et al., 2017). In some of these studies reporting lifespan extension by autophagy activation, the role of autophagy has been specifically determined by abolishing the anti-aging effects via knockdown of essential autophagy genes (Madeo et al., 2015; Nakamura and Yoshimori, 2018).

## Conclusion

The methodologies for measuring autophagy have evolved over the past decade and it is now feasible to undertake high-throughput chemical screens for identifying modulators of autophagic flux. A number of pharmacological modulators of autophagy have been identified via screening approaches or individual studies; some of which have been demonstrated to exert therapeutic benefits in diverse human diseases. Most of the key autophagy modulators have been identified either by the GFP-LC3 screening method in HeLa cells or via assessing the clearance of aggregation-prone proteins in inducible PC12 cell lines. While analysis of changes in autophagosome number with GFP-LC3 reporter requires shorter treatment period (such as 8–24 h), analysis of clearance of aggregation-prone proteins requires longer treatment duration (such as 24–72 h) depending on the nature of the transgene product. Following the primary screen, it is pertinent to characterize the high-confidence screen hits with secondary autophagy assays because there are no single assays to determine autophagic flux. These normally include analysis of autophagosome formation with bafilomycin A_1_ via immunoblotting with anti-LC3 antibody, analysis of autophagosome maturation with mRFP-GFP-LC3 reporter, and analysis of autophagy substrate (p62) clearance via immunoblotting with anti-p62 antibody (Mizushima et al., 2010; Klionsky et al., 2016).

Although the methods described in this review are those that have been generally used in the field, alternative autophagy assays could also be employed for chemical screening. One potential approach is the use of Keima, a fluorescent acid-stable protein that exhibits bimodal excitation spectra in neutral and acidic pH, such as in autophagosomes and autolysosomes, respectively (Katayama et al., 2011). The cumulative fluorescence readout can be used to measure bulk autophagic flux. This protein can also be utilized for selective autophagic flux, such as with mitochondria-targeted Keima to measure mitophagy (Katayama et al., 2011; Sun et al., 2017). However, Keima-based assays solely depend upon the lysosomal acidity and thus cannot be performed in fixed cells where the pH gradient across lysosomal membranes is lost. In addition, other screening approaches could be based on fluorescent-tagged early markers of autophagy initiation, such as with WIPI-1 (Proikas-Cezanne and Pfisterer, 2009) and DFCP1 (Axe et al., 2008); however, these methods will not capture the late events of autophagy pathway involving autophagosome maturation and cargo degradation.

For the therapeutic exploitation of autophagy modulators, mTOR-independent autophagy inducers are generally favorable and considered to have lesser side-effects than the mTOR inhibitors like rapamycin. This is because mTOR controls vital cellular functions like cell growth and translation and thus its inhibition can lead to undesirable side-effects unrelated to autophagy induction. For clinical translation to patients, it is important to determine the efficacy and penetrance of the autophagy modulators in the target organs. Future directions could include identifying specific inducers of autophagy acting at the level of autophagic machinery rather than the upstream signaling pathways.

## Author Contributions

PP, AF, SV, DK, and SS wrote the manuscript. ES and SS made the figures. PP and SS made the tables. PP, AF, SV, ES, VS, MC, PD, JT, TR, DK, and SS reviewed the manuscript.

## Conflict of Interest Statement

The authors declare that the research was conducted in the absence of any commercial or financial relationships that could be construed as a potential conflict of interest.
